# Microbial Modulation: Unraveling the Influence of Gut Microbiota on Macrophage Polarization in Tumor Microenvironments

**DOI:** 10.3390/cells15020136

**Published:** 2026-01-12

**Authors:** Jonathan Trejo, Hayes Koegeboehn, Farah Faizuddin, Ryan Logan, Michel Toutoungy, Aadil Sheikh, Tamer E. Fandy, Sergio Saucedo, Victor M. Vasquez, Thien Nguyen, Jennifer T. Grier, Ghislaine Mayer, Jessica Chacon

**Affiliations:** 1 Foster School of Medicine, Texas Tech University Health Sciences Center El Paso, El Paso, TX 79905, USAtamer.fandy@ttuhsc.edu (T.E.F.);; 2 Department of Medical Education, Texas Tech University Health Sciences Center El Paso, 130 Rick Francis Street, El Paso, TX 79905, USA; 3 School of Medicine Greenville, University of South Carolina, Greenville, SC 29605, USAjgrier@greenvillemed.sc.edu (J.T.G.)

**Keywords:** tumor-associated macrophages, gut microbiota, macrophage polarization, microbial metabolites, cancer immunotherapy

## Abstract

The intricate interplay between the human microbiota and the immune system has garnered significant attention in recent years, particularly concerning its implications in cancer biology. Macrophages, pivotal players in the tumor microenvironment (TME), exhibit diverse phenotypes that can either promote tumor progression or inhibit it. This review explores the multifaceted role of the microbiota in modulating macrophage polarization within the TME. We highlight recent findings that demonstrate how specific microbial communities influence macrophage behavior through metabolic pathways, immune signaling, and epigenetic modifications. Furthermore, we discuss the therapeutic potential of manipulating the microbiota to reprogram macrophage phenotypes, thereby enhancing antitumor immunity. By integrating insights from microbiology, immunology, and oncology, this article aims to provide a comprehensive overview of the microbiota’s impact on macrophage dynamics in cancer, paving the way for innovative therapeutic strategies that harness this relationship for improved clinical outcomes.

## 1. Introduction

### 1.1. Overview of the Microbiota and Microbiome

The microbiome is composed of trillions of bacteria, archaea, fungi, and other microorganisms that reside on various surfaces of the human body such as the gastrointestinal tract, the skin, urogenital tract, and respiratory tract [1]. Their symbiotic relationships contribute to host physiology by producing metabolites, reinforcing epithelial barriers, modulating nutrition, and shaping systemic immunity [2]. The most studied of these surfaces is the gastrointestinal tract, which exhibits a great diversity of species that increases through childhood and is maintained through adulthood before decreasing in older age [3]. This decline has been linked to the compromise of mucosal barriers with leakage of cytokines, leading to chronic inflammatory states [3]. Healthy microbiomes tend to be taxonomically conserved across humans but experience variation at the individual level. This balance of the normal microbiota can be interrupted by interventions such as diet, medications, the environment, or even geography [4]. Disruptions to the microbial community structure have been implicated in a variety of disease processes, including cardiovascular diseases (CVD), inflammatory bowel disease (IBD), dementia, and cancer, often through their effects on components of the immune system [5,6,7].

### 1.2. The Microbiota and the Tumor Microenvironment

The microbiome has been studied extensively in cancer as an oncogenic factor and key player in growth, metastasis, and immune modulation through its effects on the tumor microenvironment (TME). The TME is a dynamic and multifaceted entity that includes immune cells, stromal cells, blood vessels, and the extracellular matrix [8]. Dysregulation of the gut microbiota has been implicated in disruption of the single-cell-layer mucosal surface, promoting cytokine-driven inflammatory states and contributing to genetic mutations and carcinogenesis [9]. An important and emerging dimension of microbiota–TME interactions involves the tumor suppressor p53, a master regulator of cellular metabolism, DNA repair, and inflammation [10].

Inflammatory macrophage polarization is supported by a shift toward aerobic glycolysis, driven in part by HIF-1α stabilization and PKM2-dependent transcriptional regulation of pro-inflammatory genes. Microbiota-derived metabolites such as butyrate, lactate, and tryptophan derivatives modulate glycolytic flux and HIF-1α activity through epigenetic, GPCR-mediated, and redox-sensitive mechanisms, thereby shaping macrophage inflammatory outputs.

Oxidative phosphorylation and intact mitochondrial metabolism are hallmarks of anti-inflammatory macrophage phenotypes, supporting sustained ATP production, controlled ROS signaling, and resolution-associated gene programs. Microbial bile acids and other microbiota-derived signals regulate mitochondrial function through nuclear receptor and FXR/TGR5 pathways, linking microbial ecology to macrophage metabolic fitness and immune homeostasis.

Fatty acid oxidation (FAO) promotes regulatory and tissue-reparative macrophage states through transcriptional control by PPAR-γ and the mitochondrial biogenesis factor PGC-1β. Short-chain fatty acids and secondary bile acids enhance FAO-dependent polarization by activating lipid-sensing receptors and metabolic enzymes, integrating microbial lipid metabolism with macrophage inflammatory programming.

Although our review primarily focuses on macrophage polarization, it is important to recognize that p53 signaling is bidirectionally influenced by gut microbiota. Studies have shown that gut dysbiosis can impair wild-type (WT) p53 function, potentially compromising its tumor-suppressive role. Conversely, mutant p53 has been associated with microbiota alterations that support tumorigenesis and chronic inflammation. For instance, WT p53 contributes to maintaining intestinal barrier integrity, while mutant forms may foster microbial communities that enhance carcinogenic potential. These interactions may also indirectly shape immune cell behavior within the TME, including macrophage polarization, by modulating local inflammatory and metabolic cues. Incorporating p53 status into our understanding of microbiota–immune dynamics adds an important layer of complexity to tumor–host interactions and may influence responses to immunotherapy and metabolic interventions.

### 1.3. Focus of the Review: Microbiota and Macrophage Polarization

**Figure 1:**
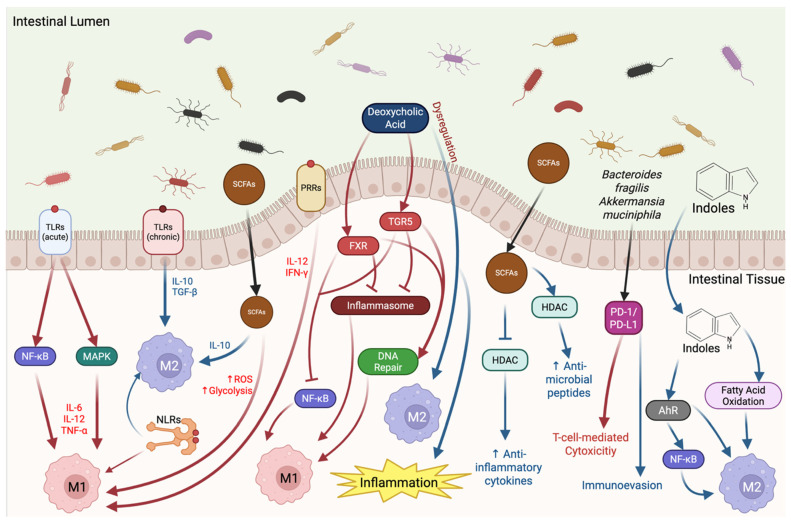
Conceptual diagram of the microbiota–macrophage–cancer axis. An overview graphic showing how gut- or tumor-associated microbiota influence macrophage phenotypes in the tumor microenvironment via immune, metabolic, and epigenetic pathways. SCFAs: short-chain fatty acids; PRRs: pattern recognition receptors; TLRs: toll-like receptors; NLRs: NOD-like receptors; AhR: aryl hydrocarbon receptor; MAPK: mitogen-activated protein kinase; HDAC: histone deacetylase; TGR5: G protein-coupled bile acid receptor 1 (also known as GPBAR1); FXR: farnesoid X receptor. Created in BioRender. Sheikh, A. (2025) https://BioRender.com/mntqj62 (accessed on 29 June 2025).

This review focuses on the emerging role of the microbiota in regulating macrophage polarization within the TME, which is critical in cancer immunobiology. Macrophages are one of the most abundant cells in the TME and exhibit plasticity switching between pro-inflammatory (M1) and anti-inflammatory (M2) phenotypes in response to environmental stimuli [11]. As demonstrated in Figure 1, the microbiota is a regulator in this phenotypic switching. Microbially derived metabolites such as short-chain fatty acids (SCFAs) and lipopolysaccharides (LPSs) contribute to macrophage behavior by activating signaling pathways like NF-κB, STAT3, and mTOR, which can drive context-dependent immunostimulatory or immunosuppressive functions [1].

Furthermore, the microbiota exerts indirect effects on macrophage activity by modulating systemic immune tone and reshaping the cytokine environment within tumors. For example, some species have been shown to promote TGF-β or IL-10 production, which would support M2 polarization, while others stimulate IFN-γ and IL-12 production, favoring the M1 pathway. Additionally, microbial signals have been shown to induce remodeling of macrophages by altering histone acetylation and DNA methylation, leading to long-term changes in gene expression [11].

All of these factors position the microbiota as an active and dynamic regulator of macrophage phenotypes within the TME. Gaining an understanding of how microbial signals play a key role in macrophage polarization could open up a promising frontier in immunotherapy, where targeted manipulation of the microbiome could aid in reprogramming macrophages towards antitumor functions, thereby strengthening therapy efficacy and improving clinical outcomes.

## 2. Macrophages in the Tumor Microenvironment

### 2.1. Macrophage Polarization Paradigm

Macrophages are remarkable plastic immune cells capable of adopting diverse activation states in response to microenvironmental cues. Traditionally, macrophage polarization is simplified into two extremes, with macrophages transitioning from an undifferentiated M0 state to either a classically activated M1 or an alternatively activated M2 phenotype [12]. Specific signals induce each phenotype and carry out specialized functions in immunity (Table 1).

### 2.2. Functional Characteristics of M1 and M2 Macrophages

#### 2.2.1. M1 Macrophages—Tumoricidal and Immunostimulatory

M1 macrophages are induced by pro-inflammatory stimuli, such as IFN-γ and toll-like receptor (TLR) ligands (e.g., TLR-4, like lipopolysaccharide (LPS)). As a result, M1 macrophages acquire a pro-inflammatory, tumor-inhibiting phenotype. They produce high levels of inflammatory cytokines (e.g., IL-1β, IL-6, IL-12, IL-23, TNF-α) and effector molecules like inducible nitric oxide synthase (iNOS/NOS2) and reactive oxygen species (ROS) [11,13,14]. M1 macrophages also upregulate antigen-presenting machinery, notably MHC-II and co-stimulatory molecules (CD80/CD86), providing them with strong T-cell activation capacity and intrinsic phagocytosis [13].

#### 2.2.2. M2 Macrophages—Immunosuppressive and Tumor-Promoting

M2 polarization occurs in the presence of anti-inflammatory, Th2-associated signals such as IL-4, IL-10, or IL-13. M2 macrophages assume an immunosuppressive, tissue-remodeling phenotype generally associated with tumor promotion and wound healing. They secrete high levels of anti-inflammatory and pro-fibrotic factors (e.g., IL-10 and TGF-β) and also express enzymes like arginase-1 (Arg1) that drive polyamine production for tissue repair and tumor growth [13]. In addition, M2 cells often express markers such as the mannose receptor (CD206) and scavenger receptor (CD163) involved in debris clearance and resolution of inflammation [15,16]. The M2 polarization cytokine profile is essentially the inverse of M1: a low IL-12 and a high IL-10 phenotype, often accompanied by elevated IL-1 receptor antagonists and decoy receptors that dampen inflammation [17]. Consequently, M2-polarized macrophages contribute to immunosuppressive, tumor-supportive functions in the tissue microenvironment.

### 2.3. Transcriptional Regulation and Plasticity

Distinct signaling pathways and transcriptional factors underlie macrophages’ polarization into M1 or M2 states. For example, M1 polarization is driven by pathways activated downstream of IFN-γ and TLR signals (e.g., LPS), which engage transcriptional factors like STAT1 and NF-kB, inducing genes like NOS2 (iNOS) and other pro-inflammatory mediators [14]. On the contrary, IL-4/IL-13 in M2 macrophages activate JAK-STAT6 and other factors like IRF4 and PPAR-γ to induce Arg1, MRC1, and other M2 genes [12]. It is crucial to note that macrophage polarization is not a rigid binary between M1 and M2, but rather a continuum. Intermediate phenotypes can co-exist in vivo, and M2 itself encompasses a variety of subtypes (e.g., M2a, M2b, M2c, M2d) that all share the same immunosuppressive properties, a high IL-10/low IL-12 profile, but differ in specific triggers and functions [13]. This plasticity allows macrophages to switch their phenotype in response to microenvironmental signals. In the context of cancer, tumor-associated macrophages (TAMs) comprise a heterogeneous mix of these different activation states, generally trending toward an M2-like, protumoral profile [18]. In summary, the M1/M2 framework is a useful tool, but actual macrophage behavior in tissues reflects a spectrum of activation states modulated by the local microenvironment.

### 2.4. Tumor-Associated Macrophages (TAMs) and Dynamic Polarization

Tumor-associated macrophages (TAMs) are among the most abundant immune cells in the tumor microenvironment (TME), often comprising 30–50% of the tumor’s stromal cell population [19]. At the earliest stage of a tumor, macrophages polarize to M1 to generate an antitumor response. These M1 macrophages release pro-inflammatory cytokines (TNF-α, IL-1β, IL-6, IL-12) and generate reactive oxygen/nitrogen species to attack tumor cells and recruit lymphocytes. The combined activities of these macrophages contribute to the recruitment and activation of cytotoxic lymphocytes, including CD8^+^ T cells and natural killer (NK) cells, to attack and eliminate tumor cells. The M1 phenotype is thus associated with a tumoricidal, immunostimulatory profile that restrains early tumor growth and facilitates immune surveillance.

This initial response can inhibit emerging cancer cells; however, as the tumor progresses and the microenvironment evolves, a dramatic shift occurs in macrophage polarization. The chronic inflammation eventually leads to the production of anti-inflammatory signals and the transition from M1 to M2 macrophages to prevent excess inflammation from happening. Studies have shown that in advanced tumors, TAMs predominantly acquire an M2-like phenotype, which is actively favored by the TME [18,20].

### 2.5. Microenvironmental Influences on TAM Polarization

Tumor-derived signals (discussed below) allow the infiltrating macrophages to switch from an M1 (tumoricidal) state to an M2 (tumor-promoting) state, thereby flipping an early antitumor immune reaction into a protumor macrophage [11,21]. As a result, what starts as an effective tumoricidal response can be changed into a response that supports tumor growth, which catalyzes the progression and malignancy of the tumor. Notably, the TME is known to predominantly favor an M2 phenotype [22]. Therefore, TAMs usually refer to M2 macrophages.

The TME plays a pivotal role in determining the functional state of TAMs. Multiple signals, including cytokines, hypoxia, metabolites, and immune cell crosstalk, contribute to whether infiltrating macrophages adopt a classically activated M1 phenotype or an alternatively activated M2 phenotype [23,24]. By the time most tumors are well established, the balance of these factors typically favors M2 polarization, enabling TAMs to support tumor growth and immune evasion [25]. The following subsections explore the major influences shaping TAM polarization within the TME.

#### 2.5.1. Cytokines and Growth Factors

The local balance of Th1 versus Th2 signals is a critical determinant of macrophage polarization. Tumor and stromal cells in the TME frequently produce Th2-type and immunosuppressive cytokines, such as IL-4, IL-13, IL-10, and TGF-β, which push macrophages toward an M2 state. IL-4 and IL-13 (often secreted by Th2 cells or cancer-associated fibroblasts) activate the STAT6 signaling pathway, including M2-specific genes. IL-10, produced by Tregs, tumor cells, or even the macrophages themselves in an autocrine loop, activates STAT3, reinforcing an M2, anti-inflammatory cascade. Tumor-derived CSF-1 (M-CSF) further recruits monocytes and promotes their differentiation into M2-like TAMs [12]. Conversely, pro-inflammatory Th1-type cytokines like IFN-γ or granulocyte-macrophage colony-stimulating factor (GM-CSF), which would favor M1 polarization, are often scarce in the TME due to tumor-driven immune evasion. As a result, the cytokine environment in most tumors heavily skews TAMs toward a protumoral M2 phenotype [20].

#### 2.5.2. Hypoxia and Metabolic Stress

Hypoxia is a hallmark of solid tumors and exerts a strong influence on macrophage polarization. Hypoxic tumor cells secrete chemotactic factors like VEGF, CCL2, CSF-1, and endothelins to attract monocytes/macrophages into oxygen-deprived regions [26,27]. Upon entry, these cells encounter low oxygen tension, which stabilizes hypoxia-inducible factors (HIF-1α and HIF-2α) [28]. These transcription factors activate genes that promote the M2 phenotype, including those involved in angiogenesis (e.g., VEGF), matrix remodeling (e.g., Arg-1), and immune suppression (e.g., PD-L1). In parallel, hypoxia is linked to the accumulation of lactic acid and extracellular acidosis in the TME (the Warburg effect). Lactic acid has been shown to downregulate NF-kB signaling and induce M2-associated gene expression via HIF-1α [28]. In essence, the metabolic environment, characterized by hypoxia, high lactate, low pH, and nutrient competition, conditions macrophages to adopt an M2/oxidative metabolism that aligns with tissue remodeling and immune suppression, rather than an M1/glycolytic, inflammatory state [12].

#### 2.5.3. Tumor-Derived Molecular Signals

Additionally, tumor cells often shed danger-associated molecular patterns (DAMPs) and immunomodulatory metabolites like adenosine, both of which skew macrophage polarization. Adenosine accumulates under hypoxic conditions and binds to A2A receptors on macrophages, reducing pro-inflammatory cytokine production and favoring an M2 state [8]. Similarly, prostaglandin E2 (PGE2) released by tumor cells acts to suppress IL-12 and promotes IL-10 production in macrophages, further reinforcing the M2-like state.

#### 2.5.4. Immune Cell Interactions

Cell–cell interactions within the TME also shape macrophage behavior. Tegs secrete IL-10 and TGF-β in the tumor, reinforcing M2 polarization of TAMs. In turn, TAMs recruit additional Tregs, establishing a positive feedback loop of immunosuppression. Conversely, the presence of Th1-biased CD4+ T cells or activated NK cells that produce IFN-γ can push macrophages toward an M1 phenotype; however, many tumors actively exclude and/or suppress these IFN-γ sources. Tumor-associated neutrophils and MDSCs can release IL-10 or Arg-1, which also influence macrophages toward M2 [13]. Even the ECM can influence macrophages: for example, high extracellular levels of IL-1 from chronic inflammation may initially promote M1 activation, but sustained exposure ultimately leads to a compensatory M2 reprogramming.

### 2.6. Protumoral Functions of M2-Polarized TAMs

#### 2.6.1. Promotion of Invasion and Metastasis

Once polarized to the M2 phenotype, TAMs become the key component of tumor progression and malignancy. They engage in cascade-like interactions within the TME, meaning local, interconnected feedback loops that accelerate tumor aggressiveness and metastasis. A well-known positive feedback loop is the Endothelial Growth Factor–Colony Stimulating Factor-1 (EGF-CSF-1) circuit: Tumor cells release CSF-1 to recruit and differentiate macrophages, while TAMs secrete EGF, which enhances tumor cell migration and invasion. M2 TAMs also secrete IL-6 and IL-8, promoting epithelial–mesenchymal transition (EMT) and tumor cell motility [29]. To enable physical invasion, TAMs also release matrix-degrading enzymes (MMPs) (e.g., MMP-1, MMP-2, MMP-9), cathepsins, and urokinase plasminogen activator (uPA) that specifically break down the extracellular matrix (ECM), which helps the tumor invade surrounding tissue. This proteolysis clears a path for tumor cells to disseminate into surrounding tissues and, eventually, to intravasate into blood vessels. Simultaneously, M2 TAMs promote angiogenesis by secreting an abundance of growth factors and pro-angiogenic cytokines (EGF, PDGF, TGF-β, FGF, IL-8) that feed the growing tumor and collectively promote malignancy. The resulting vasculature is typically disorganized and leaky, providing both nutrients and easy escape routes for circulating tumor cells.

Importantly, studies in animal models have shown that depleting TAMs can significantly impair tumor vascularization and reduce metastatic spread. These findings underscore the essential role of TAMs, particularly M2-polarized subsets, in driving tumor angiogenesis and metastatic progression [18]. Additionally, TAM-derived proteases (MMPs) and chemokines like CCL5, CCL18, and S100 recruit fibroblasts and additional stromal support, further enhancing tumor invasion. The cumulative effect of all these factors is a multifaceted promotion of tumor invasion that paves the way for tumor cells by remodeling the matrix and guiding them towards the blood vessel for dissemination.

#### 2.6.2. Immune Suppression and Evasion

Beyond the physical aspects of tumor growth, M2-polarized TAMs play a central role in immune evasion, allowing cancer to thrive unchecked. TAMs secrete chemokines such as CCL2 and CCL22, which attract immunosuppressive populations, such as myeloid-derived suppressor cells (MDSCs) and regulatory T cells (Tregs), into the tumor [30,31]. The resulting tumor microenvironment becomes hostile to cytotoxic T lymphocytes (CTLs) and natural killer (NK) cells, weakening the immune system’s ability to attack the tumor.

Importantly, multiple studies using animal models have demonstrated that selective depletion of TAMs leads to a marked reduction in Treg infiltration and a corresponding slowdown in tumor growth. These findings provide compelling in vivo evidence that TAM-mediated recruitment of suppressor cells, particularly through CCL2 signaling, is a critical mechanism by which tumors evade immune destruction [12].

TAMs also directly dampen T cells and natural killer (NK) cells through the release of immunosuppressive cytokines and the expression of inhibitory ligands. IL-10 inhibits Th1 and CTL effector functions and promotes Treg development, while TGF-β can arrest CTL and NK cell cytotoxicity and induce differentiation of additional Tregs [11]. Elevated levels of these TAM-derived cytokines create a tolerant immune environment, effectively “shielding” the tumor from immune attack. Moreover, TAMs frequently express immune checkpoint ligands such as PD-L1 on their surface, which bind to PD-1 on T cells, causing functional exhaustion [12,18]. High levels of PD-L1 TAMs have been correlated with poor T cell infiltration and can contribute to resistance against PD-1/PD-L1 checkpoint inhibitors [18].

#### 2.6.3. TAM Functions Across Gastrointestinal Cancers

Tumor-associated macrophages (TAMs) are critically involved in the pathogenesis of multiple gastrointestinal (GI) cancers, where they adopt cancer type-specific functions shaped by local metabolic and microbial influences. In colorectal cancer (CRC), TAMs frequently polarize toward an M2-like, immunosuppressive state that promotes angiogenesis, epithelial–mesenchymal transition, and metastasis, in line with extensive evidence linking M2 TAM enrichment to poor outcomes across solid tumors [31]. CRC-associated microbes, such as *Fusobacterium nucleatum*, further enhance macrophage recruitment and pro-inflammatory signaling via NF-κB activation, reinforcing tumor-permissive inflammation within the colonic microenvironment [32]. In gastric cancer, chronic microbially driven inflammation—most notably that associated with *Helicobacter pylori* infection—promotes sustained macrophage activation and skewing toward M2 phenotypes that support tissue remodeling and tumor progression [33]. Pancreatic ductal adenocarcinoma (PDAC) similarly shows dense TAM infiltration; within this TME, microbial-derived tryptophan metabolites foster Aryl hydrocarbon receptor (AhR)-dependent immunoregulatory macrophage polarization, consistent with the broader role of tryptophan catabolism in driving M2-like TAM phenotypes [34,35]. Collectively, these observations underscore that while TAM-mediated immune suppression is a shared hallmark across GI malignancies, the specific microbial, cytokine, and metabolic cues shaping macrophage behavior vary by tumor type, highlighting the need for GI cancer-specific, TAM-targeted therapeutic strategies.

### 2.7. Clinical Relevance of TAM Infiltration

Clinical studies across multiple cancer types have consistently shown that a high density of TAMs, particularly those exhibiting an M2-like phenotype, is strongly associated with advanced tumor stage, increased angiogenesis, metastasis, and poor patient outcomes [31]. Meta-analyses involving tens of thousands of patients have reinforced these findings, demonstrating that elevated M2 TAM infiltration broadly correlates with unfavorable prognosis in cancers such as breast, ovarian, colon, and lung [31]. In contrast, tumors with higher levels of M1-like macrophage infiltration are generally linked to better clinical outcomes. These observations not only highlight the protumoral role of M2 TAMs but also suggest that reprogramming their phenotype could be a promising therapeutic strategy.

### 2.8. Summary

In summary, through cytokine-mediated immunosuppression, recruitment of suppressor cells, and checkpoint inhibition, M2 TAMs establish an immunosuppressive niche that allows tumors to grow relatively unchecked by the immune system. The immunosuppressive role of TAMs not only fosters tumor progression but also poses a barrier to immunotherapies and traditional treatments. The TME provides a rich, complex network of cytokines, metabolic conditions, molecular signals, and intercellular communication that collectively drive macrophages toward a tumor-promoting M2 phenotype. Understanding these factors is crucial, as emerging therapies aim to interrupt these signals and reprogram TAMs back to a tumoricidal M1 state to enhance treatment outcomes.

## 3. Microbiota as Modulators of Macrophage Polarization

The gut microbiota plays a critical role in shaping macrophage polarization within the TME by producing a variety of metabolites that regulate immune cell function and metabolic pathways [34,36] (Table 2). These microbial metabolites act as essential communicators between host and microbes, influencing macrophage phenotypes and thereby modulating tumor immunity [35,37,38,39]. Deciphering these mechanisms is pivotal for understanding the complex immunometabolic milieu that affects cancer progression and therapy responsiveness.

### 3.1. Microbial Metabolites That Influence Macrophage Polarization

#### 3.1.1. Short-Chain Fatty Acids (SCFAs)

Short-chain fatty acids (SCFAs), including acetate, propionate, and butyrate, are generated primarily via bacterial fermentation of dietary fibers in the colon. They exert immunoregulatory effects by activating G-protein-coupled receptors (e.g., GPR41, GPR43) and inhibiting histone deacetylases, thereby altering gene expression profiles in macrophages [38,39].

The impact of SCFAs is intricately shaped by the tissue-specific characteristics of the tumor microenvironment (TME). SCFAs such as butyrate promote M1-like, pro-inflammatory macrophage polarization that supports antitumor immunity, as demonstrated in colorectal cancer models, where butyrate increases glycolysis and reactive oxygen species (ROS) in tumor-associated macrophages (TAMs), enhancing their tumoricidal functions (26). In other cancer contexts such as prostate cancer, elevated SCFA levels contribute to tumor progression by fostering M2-like macrophage polarization through mechanisms involving autophagy and chemokine secretion, illustrating the context-dependent effects of these metabolites [34,40]. These divergent effects are modulated by several factors, including local oxygen levels, lactate accumulation, cytokine composition, and immune cell infiltration patterns. In lung or prostate tumors, SCFAs may promote M2 polarization and immunosuppression. These divergent effects are modulated by several factors, including local oxygen levels (hypoxia promotes M2), lactate accumulation, cytokine composition (e.g., IFN-γ vs. IL-10), and immune cell infiltration patterns. For instance, butyrate may enhance oxidative phosphorylation in M2 macrophages within hypoxic prostate tumors while promoting glycolysis in colon-associated TAMs.

Context-dependent polarization of macrophages are present under a variety of physiological conditions. Of note, pregnancy-specific shifts in gut microbiota are associated with elevated SCFA levels that support low-grade inflammation and increased M2 macrophage polarization in maternal tissues, facilitating immune tolerance of the fetus [41,42]. This immunoregulatory role of SCFAs during pregnancy highlights a broader systemic impact of microbial metabolites on immune tone and macrophage phenotype, with potential implications for understanding immune adaptation in cancer and chronic inflammatory states.

Insulin resistance represents another important immunometabolic state linked to alterations in gut microbiota and SCFA production. Individuals with insulin resistance frequently exhibit dysbiosis characterized by a reduction in SCFA-producing bacteria and increased intestinal permeability. This dysbiotic shift promotes systemic low-grade inflammation, favoring M1 macrophage polarization in adipose tissue and the tumor microenvironment [43,44]. Insulin-resistant states are associated with elevated levels of pro-inflammatory cytokines such as IL-6 and TNF-α, contributing to macrophage-mediated immune activation [43,44]. In contrast, some studies report compensatory IL-10 production and M2-like markers in adipose tissue macrophages under chronic hyperglycemia, suggesting a context-dependent duality [43,44]. Moreover, microbial modulation of bile acid metabolism and downstream activation of farnesoid X receptor (FXR) signaling also influences insulin sensitivity and macrophage phenotype, further linking gut microbial metabolites to systemic immunometabolic networks involved in both metabolic syndrome and tumor progression [45].

Clinically, diminished SCFA levels have been linked to poor prognosis in colorectal and pancreatic cancers, emphasizing the therapeutic potential of microbiota-targeted interventions such as fiber-rich diets and probiotics to restore SCFA production [36,37,38]. These findings underscore the necessity of understanding the metabolic and immunologic characteristics of individual TMEs when considering microbiota-based interventions.

#### 3.1.2. Tryptophan Metabolites

Gut microbial metabolism of tryptophan produces indole derivatives that activate the aryl hydrocarbon receptor (AhR) on macrophages, modulating their immunosuppressive functions [34,35]. AhR activation upregulates anti-inflammatory cytokines such as IL-10 and immune checkpoint molecules while inhibiting NF-κB signaling, promoting M2-like macrophage phenotypes within the TME [35]. Elevated tryptophan catabolite production correlates with immunosuppressive macrophage infiltration and tumor progression in pancreatic ductal adenocarcinoma (PDAC) [35,36]. Pharmacologic targeting of AhR signaling reprograms TAMs towards antitumor phenotypes in preclinical models, highlighting this axis as a promising therapeutic target [34].

Interestingly, microbiota-driven tryptophan metabolism is also modulated during pregnancy, contributing to increased systemic levels of indole derivatives that promote M2 macrophage polarization and immune tolerance [45]. This parallel suggests that the mechanisms facilitating fetal tolerance may be repurposed in the tumor microenvironment to enable immune evasion, underscoring the importance of contextual and temporal factors in microbial metabolite signaling.

#### 3.1.3. Bile Acids

Microbial transformation of primary bile acids into secondary bile acids such as deoxycholic acid modulates macrophage activity via nuclear receptors, including the farnesoid X receptor (FXR) and TGR5 [37]. Activation of these receptors attenuates pro-inflammatory signaling by suppressing NF-κB and inflammasome pathways and promotes DNA repair mechanisms [37]. Dysregulation of bile acid metabolism exacerbates inflammation and M2 macrophage recruitment in colitis-associated colorectal cancer, implicating impaired FXR signaling in carcinogenesis [37]. The diverse effects of bile acids on macrophage function depend on local metabolite concentrations and receptor interactions, contributing to complex immunoregulatory roles in the TME [46].

#### 3.1.4. Dietary Regulation of GI Microbiota and Its Consequences for Macrophage Programming in the Tumor Microenvironment

Diet is one of the most powerful modulators of the gastrointestinal microbiota and plays a central role in shaping macrophage function within the tumor microenvironment (TME). Dietary components, including fiber, fat, protein, micronutrients, and simple sugars, directly influence microbial community structure, metabolite production, and inflammatory signaling, ultimately impacting macrophage polarization and tumor immunity.

High-fiber diets, rich in fermentable complex carbohydrates, promote the growth of SCFA-producing taxa such as *Clostridium*, *Faecalibacterium*, and *Bacteroides* [37,38,39]. The resulting increases in SCFAs, such as butyrate, propionate, and acetate, exert potent immunomodulatory effects by functioning as metabolic substrates and epigenetic regulators in macrophages. Butyrate enhances histone acetylation, modulates chromatin accessibility, and can drive either M1- or M2-like macrophage phenotypes depending on the tumor context [38,39]. In colorectal cancer, elevated luminal butyrate supports antitumor M1 polarization through enhanced glycolysis and ROS generation [38], whereas in prostate cancer, increased SCFA flux promotes M2-like macrophage recruitment and tumor progression through TLR3 signaling and autophagy induction [47]. These observations underscore the importance of tissue-specific metabolic and cytokine environments in determining SCFA effects on TAMs.

Beyond metabolites, dietary fiber alters cytokine landscapes through microbiota-dependent pathways. In vitro gut-on-a-chip models demonstrate that fiber fermentation can augment macrophage release of IL-1β, IFN-γ, TNF-α, IL-12p70, and IL-23 in the presence of colorectal tumor cells, reinforcing pro-inflammatory M1 pathways [32]. Such findings highlight that diet-driven microbial metabolism can modulate early inflammatory cues that shape macrophage fate in the TME.

In contrast, high-fat and high-sugar diets promote dysbiosis characterized by decreased SCFA producers, increased intestinal permeability, and translocation of microbial LPSs into circulation, an immune metabolic state linked to chronic activation of TLR4–NF-κB signaling and M1 skewing in peripheral and adipose tissue macrophages [43,44]. Paradoxically, prolonged exposure to lipotoxic and glucose-rich environments may eventually drive compensatory M2-like polarization, illustrating the metabolic plasticity of macrophages in chronic inflammatory states. These metabolic signatures influence systemic immune tone and can predispose the TME to immunosuppressive myeloid infiltration.

Dietary fat-driven alterations in bile acid pools also affect macrophage behavior through FXR and TGR5 signaling. High-fat diets elevate secondary bile acids that suppress NLRP3 inflammasome activation and promote oxidative phosphorylation-dependent M2 macrophage polarization, contributing to immunosuppressive TMEs in GI cancers [37,45]. Similarly, diets rich in tryptophan enhance microbial production of indole derivatives, which activate AhR and reinforce immunoregulatory M2-like TAM phenotypes in pancreatic and colorectal cancers [34,35].

Importantly, diet can indirectly regulate macrophage metabolism by altering tumor-derived nutrients and systemic metabolite pools. Tumor-associated macrophages rely on fatty acid oxidation and mitochondrial respiration for M2-like functions; high-fat diets provide excess lipid substrates that may reinforce this phenotype, whereas fiber-rich diets increase SCFAs that can disrupt M2 metabolic programs or, in certain contexts, further promote them.

Finally, diet-induced microbial shifts correlate with clinical outcomes. For example, reduced SCFA-producing bacteria and elevated inflammatory metabolites are linked to poor prognosis in colorectal and pancreatic cancers, while restoration of beneficial taxa through dietary interventions enhances antitumor immunity and responsiveness to immunotherapy [36,37,48].

Collectively, these findings illustrate a dynamic and bidirectional diet–microbiota–macrophage axis in GI cancers, wherein dietary patterns sculpt microbial ecosystems and metabolite profiles that ultimately dictate macrophage polarization, inflammatory tone, and therapeutic responsiveness within the TME. Understanding these interactions provides a foundation for developing diet-based or microbiota-targeted interventions to reprogram TAMs and improve cancer outcomes.

### 3.2. Metabolic Reprogramming of Macrophages in the Tumor Microenvironment

Macrophage polarization is intricately linked to their metabolic state. M1-like macrophages predominantly utilize aerobic glycolysis to fuel pro-inflammatory and tumoricidal functions, producing intermediates such as succinate that stabilize HIF-1α and promote reactive oxygen and nitrogen species [35,48]. In contrast, M2-like TAMs depend mainly on oxidative phosphorylation and fatty acid oxidation, supporting immunosuppressive and protumoral activities [35,48].

In addition to influencing core metabolic pathways, microbial metabolites also affect redox homeostasis in macrophages. For example, butyrate has been shown to enhance ROS production in M1 macrophages in colorectal cancer (see Section 3.1.1), thereby contributing to their pro-inflammatory activity. Moreover, depletion of intracellular glutathione (GSH), a key antioxidant, is associated with M1 polarization and enhanced antitumor responses. Conversely, elevated GSH levels support an M2-like phenotype by maintaining redox balance, particularly in hypoxic tumor microenvironments (TMEs). The gut microbiota plays a crucial role in this redox regulation by modulating host GSH biosynthesis through control of cysteine and methionine availability. This metabolic and redox modulation influences cytokine secretion profiles, angiogenesis, and responses to immunotherapies [40,48].

Dietary sugars also represent an important but under-discussed factor in macrophage metabolic reprogramming. High glucose and fructose intake has been shown to alter gut microbiota composition, often reducing SCFA-producing taxa and increasing intestinal permeability and lipopolysaccharide (LPS) translocation. These changes can stimulate M1 macrophage polarization via Toll-like receptor 4 (TLR4) signaling and ROS generation. However, chronic high-sugar diets have also been associated with increased IL-10 production and M2-like markers in adipose tissue macrophages, highlighting a tissue- and context-dependent effect. By reshaping the microbiota and modifying the metabolite and endotoxin landscape, excessive sugar intake may indirectly influence macrophage polarization, TLR signaling, and inflammasome activation within the TME. These findings underscore the need to consider dietary components and microbiota-driven immunometabolism in the design of macrophage-targeted cancer therapies.

### 3.3. The Interplay of the TME and the Microbiota on Macrophage Polarization

A bidirectional interaction exists between gut microbiota and tumor metabolism. Tumor-derived factors disrupt the gut microbial community, reducing beneficial SCFA-producing bacteria and altering bile acid metabolism, which subsequently modifies circulating metabolite pools available to immune cells [48]. These systemic metabolites impact monocyte differentiation and macrophage polarization within tumors, shaping the immune landscape and influencing therapeutic outcomes [46,48].

Furthermore, the composition and functional output of the microbiota evolve alongside tumor progression. Early-stage tumors may exhibit loss of SCFA-producing taxa such as *Lachnospiraceae* due to tumor-derived factors [45], leading to reduced local M1 activation and impaired barrier function [49,50,51]. As tumors progress, selective pressures such as inflammation, hypoxia, and immune suppression may favor the enrichment of protumorigenic microbes like *Fusobacterium nucleatum* or *Prevotella* spp. [49,50,51]. These microbial shifts can directly impact macrophage polarization by altering local metabolite pools, PRR signaling (e.g., TLR4, TLR7/8 NOD2), and cytokine production [49,50,51]. Consequently, the evolving microbiota serves as both a driver and a modulator of macrophage function within the TME, further emphasizing the need for longitudinal microbial profiling in cancer management. Microbiota-derived lipoproteins, structurally diverse LPS variants, and SCFAs collectively shape TLR2/TLR4 signaling by tuning MyD88–NF-κB activation, thereby calibrating macrophage inflammatory responses toward tolerance or activation depending on the microbial context. Microbial and host RNA species activate endosomal TLR7/8, with microbiota-driven shifts in RNA abundance and composition influencing basal macrophage inflammatory tone and sensitivity to antiviral and inflammatory cues. NOD2 detection of muramyl dipeptide integrates with microbial metabolite signaling to modulate RIP2-dependent NF-κB and MAPK pathways, fine-tuning innate immune activation and inflammatory output.

### 3.4. Therapeutic Implications and Emerging Strategies

**Figure 2:**
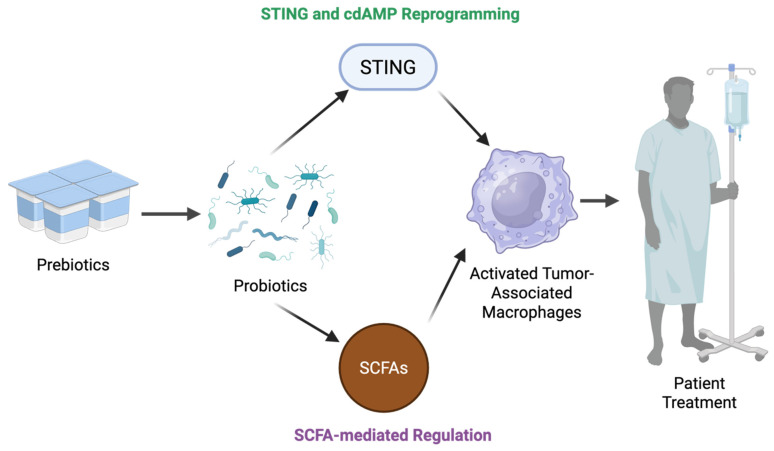
Therapeutic strategies targeting microbiota–macrophage interactions. A pipeline-style graphic or flowchart outlining therapeutic interventions (e.g., probiotics, fecal transplants, diet, epigenetic drugs) and their expected effects on TAM reprogramming and tumor immunity. Created in BioRender. Sheikh, A. (2025) https://BioRender.com/eb6yori (accessed on 29 June 2025).

Emerging therapeutic approaches targeting the microbiota—via diet, probiotics, or fecal microbiota transplantation—aim to restore favorable metabolite profiles and reprogram TAMs to enhance anti-cancer immunity [34,48]. Overall, microbiota-derived metabolites critically regulate macrophage polarization and function in the TME by modulating metabolic and signaling pathways, directly impacting tumor progression and response to therapy (Figure 2). These insights underscore the microbiota as a promising target for innovative cancer immunotherapies.

## 4. Immune Signaling Pathways Modulated by the Microbiota

### 4.1. Activation of Pattern Recognition Receptors (PRRs)

#### 4.1.1. Toll-like Receptors (TLRs) and Macrophage Polarization

The gut microbiota exerts significant influence on macrophage polarization in the TME through the activation of pattern recognition receptors (PRRs), including Toll-like receptors (TLRs) and NOD-like receptors (NLRs). These innate immune sensors recognize conserved microbial-associated molecular patterns (MAMPs) such as LPS, peptidoglycan, and flagellin. TLRs—particularly TLR2, TLR4, and TLR9—have been implicated in shaping macrophage polarization by skewing the balance between classically activated (M1-like) and alternatively activated (M2-like) phenotypes. Activation of TLRs typically triggers NF-κB and MAPK pathways, leading to the production of pro-inflammatory cytokines like IL-12, TNF-α, and IL-6 that support antitumor immunity. Conversely, chronic or dysregulated PRR signaling, often resulting from dysbiosis, can promote M2-like polarization through IL-10 and TGF-β production, contributing to immunosuppression and tumor progression.

#### 4.1.2. NOD-like Receptors (NLRs) and Inflammasome Activation

Similarly, NOD-like receptors such as NOD2 and components of the inflammasome (e.g., NLRP3) mediate the sensing of intracellular bacterial products and have been shown to influence the secretion of IL-1β and IL-18, further modulating macrophage function within the TME.

### 4.2. Microbial Regulation of Cytokine Production and Immune Checkpoints

Commensal and pathogenic microbes shape cytokine landscapes and immune checkpoint expression, thereby influencing macrophage plasticity and the broader immunological tone of the TME. Gut-derived metabolites and microbial ligands can either promote or suppress the release of key cytokines that govern macrophage polarization. Short-chain fatty acids (SCFAs), for example, can enhance IL-10 production and promote M2-like differentiation, while microbial activation of PRRs may induce IL-12 and IFN-γ to favor M1-like profiles. Furthermore, microbial modulation of cytokine signaling cascades intersects with the regulation of immune checkpoint molecules such as PD-L1. Certain bacteria, such as *Bacteroides fragilis* and *Akkermansia muciniphila*, have been shown to influence PD-1/PD-L1 axis expression in both tumor cells and infiltrating immune cells, including macrophages. This modulation can either potentiate antitumor immune responses by enhancing T cell-mediated cytotoxicity or facilitate immune evasion by reinforcing immunosuppressive circuits. Thus, the microbial imprint on cytokine dynamics and checkpoint signaling represents a critical node through which the gut microbiota can reprogram macrophage behavior and impact tumor immunity.

### 4.3. Specificity of the TME in Programming Macrophage Responses

The impact of the microbiota on macrophage responses is multidimensional and may be heavily affected by the tumor microenvironment, as varying responses are seen in the context of different tumor types. In colorectal cancer, the presence of key bacterial species, such as Fusobacterium nucleatum, promotes NF-kB signaling, which serves to recruit macrophages to the local site and promote M1 polarization (https://www.frontiersin.org/journals/immunology/articles/10.3389/fimmu.2024.1431747/full, accessed on 1 July 2025). Furthermore, dietary fiber metabolism is associated with release of inflammatory cytokines, such as L-1β, IFN-γ, TNF-α, MCP-1, IL-8, IL-12p70 and IL23, from macrophages in the presence of colorectal cancer cells [32].

Alternately, in mouse melanoma models, the tumor microenvironment was associated with macrophage dysfunction, with limited production of reactive oxygen species and decreased macrophage-mediated tumor cell death [52]. A similar immunosuppressive response is seen in macrophages in the context of prostate cancer. Specific components of the skin or tissue microbiota, such as Bacteriodes, correlated with decreased expression of TLR-3 and TGF-b. These cytokine responses reflect a polarization of macrophages towards the tumor-permissive M2 macrophage phenotype [53].

## 5. Epigenetic Regulation of Macrophage Phenotypes by Microbiota

Epigenetic modifications are heritable and reversible gene expression changes that occur without altering the DNA sequence of the genome and play an essential role in both physiological and pathological conditions [54]. These modifications involve DNA methylation, histone posttranslational modifications, chromatin remodeling, non-coding RNAs like microRNAs (miRNA), and other components of chromatin that lead to gene expression modulation [55] (Table 3). Histone posttranslational modifications include various chemical modifications like sumoylation, ubiquitylation, acetylation, phosphorylation, citrullination, crotonylation, methylation and others [56].

### 5.1. Core Epigenetic Mechanisms Affecting Gene Expression

Epigenetic changes are regulated by enzymes that add (epigenetic writers) or delete (epigenetic erasers) the chemical moiety with consequent changes in gene expression.

Alterations in these histone modifications are associated with aging, development of various cancers, and degenerative diseases [57]. DNA methylation is another epigenetic modification that contributes to essential biological processes such as X-chromosome inactivation, retrotransposon silencing, genome stability, genomic imprinting, and pathological diseases like cancer and neurodegenerative diseases [58]. DNA methylation changes at promoters and enhancers contribute to stable yet reversible macrophage phenotypic states by regulating lineage- and stimulus-specific gene expression. Microbiota-driven metabolic cues influence DNA methyltransferase and demethylase activity, enabling environmental imprinting of macrophage plasticity in response to microbial and inflammatory signals. Nonetheless, miRNAs and chromatin remodeling are other epigenetic modifications that are essential for several physiological and pathological conditions. The prominence of epigenetic modifications emerges from their reversibility and druggability.

### 5.2. Microbiota-Derived Modulation of Epigenetic Enzymes

The microbiota could influence its host physiology through multiple epigenetic mechanisms like influencing the availability of chemical donors for DNA methylation or histone modifications and by regulating the activity or expression of epigenetic modifying enzymes. For instance, the intestinal microbiota is known to generate folate and other B vitamins, including riboflavin (B2) and cobalamin (B12), which are essential nutrients that are not biosynthesized by the human body. Both folate and vitamin B12 are essential for the conversion of homocysteine into methionine, a precursor for the universal methyl donor S-adenosyl-L-methionine (SAM). Consequently, a decrease in either folate or B12 level could decrease DNA methylation with consequent gene expression changes [59]. Indeed, rats fed with methyl-deficient diets (a diet that lacks methyl donors like choline, methionine or folate) showed decreased SAM pools with consequent DNA hypomethylation of protooncogenes like c-fos and c-myc and simultaneous increase in their mRNA expression [60]. Furthermore, stimulation of the serine, glycine, one-carbon (SGOC) metabolism by L-lysine derived from the gut microbiota increased the level of SAM in dendritic cells with a subsequent increase in dimethylation of H3K79 (H3K79me2) at the gene promoters of TGF-β and Stat3, promoting immune tolerance to harmless antigens by dendritic cells [61]. Accordingly, the above examples demonstrate the role of the gut microbiota in modulating both DNA and histone methylation with consequent gene expression changes that could affect pathological or physiological conditions like tumor development and immune tolerance.

### 5.3. SCFA-Induced Histone and DNA Modifications

Short chain fatty acids (SCFAs) like butyrate and propionate derived from fiber fermentation by the gut microbiota induced gene expression changes in host cells through epigenetic mechanisms like histone acetylation and DNA methylation [62,63]. Histone acetylation is a dynamic process that is controlled by histone acetyltransferases (HATs) and histone deacetylases (HDACs), where acetylation is associated with increased transcription of genes and deacetylation is associated decreased gene transcription. Histone acetylation dynamics regulated by HDACs and HATs play a central role in controlling macrophage inflammatory gene accessibility and transcriptional plasticity. Microbiota-derived SCFAs, particularly butyrate and propionate, inhibit class I and II HDACs and shift the balance toward a more permissive chromatin state, thereby modulating NF-κB-dependent inflammatory programs and macrophage polarization. Gut microbiota-derived butyrate inhibited HDACs in macrophages and dendritic cells with consequent increase in expression of anti-inflammatory cytokines [64]. Similarly, antimicrobial peptides were upregulated by the microbiota via butyrate-induced HDAC inhibition and enhanced targeted pathogen elimination [65]. The SCFA propionate was also shown to induce promoter DNA methylation in the DAB1 gene, which is associated with lower vitamin D plasma concentrations and increased diabetes risk [62].

### 5.4. Epigenetic Specificity of Butyrate Across Tumor Types

The epigenetic activity of butyrate is highly context-dependent and varies significantly across tumor types [47,66,67]. As a potent HDAC inhibitor, butyrate can influence macrophage polarization by altering chromatin accessibility and transcription of inflammatory genes such as Nos2 and IL-12 beta [68]. In colorectal cancer, where butyrate levels are naturally elevated due to microbial fermentation in the colon [69], this activity promotes M1 macrophage polarization and antitumor immunity. The butyrate effects on macrophage-mediated antitumor immunity on colorectal cancer cells are further compounded by transcriptional up-regulation of pro-apoptotic products as a result of the histone hypoacetylation [70]. Administration of butyrate to human colorectal cancer cell lines also specifically contributed to decreased phosphorylation of the extracellular signal-regulated kinases 1 and 2 (ERK 1/2), which reduced the proliferation of the neoplastic cells [71]. A similar antitumor effect has been observed with sodium butyrate in the context of melanoma, as the HDAC inhibitor function of butyrate is thought to shift macrophage polarization away from M2 polarization and reduce the expression of macrophage-derived immunosuppressive cytokines, including IL-10 and TGF-b [72].

Alternately, in prostate cancer, butyrate has been shown to accelerate tumor progression by inducing autophagy and upregulating the chemokine CCL20 through TLR3 signaling, ultimately recruiting M2-like macrophages that support immune suppression [47].

These differences between the effects of microbial metabolites in colorectal vs. prostate cancer are likely due to tumor-specific epigenomic landscapes [73], where the microbiota adjacent to colorectal cells contributes to altered promoter methylation states, although the specific mechanisms by which this occurs have not been revealed [73]. On the other hand, hypermethylation is prevalent in prostate cancer [74]. Further variation in the epigenomic profile of these distinct tumor types may be mediated by differential expression of HDAC isoforms [75], local cytokine environments [76] and variation in butyrate transporter availability (e.g., SLC5A8) [77]. Therefore, the tumor microenvironment (TME) plays a central role in determining whether butyrate exerts pro- or antitumor effects via epigenetic mechanisms.

### 5.5. Microbiota, Chromatin Remodeling and MicroRNA–Microbiota Crosstalk

Chromatin remodeling is a dynamic process that alters chromatin configuration and DNA accessibility to transcription factors and other regulatory proteins with consequent changes in gene expression. The remodeling is mediated through covalent histone modifications, DNA methylation and ATP-dependent protein complexes that alter nucleosome positioning and is involved in several biological processes like DNA repair and replication, chromosome condensation and segregation and apoptosis [78,79]. The gut microbiota can significantly influence host chromatin remodeling through different mechanisms, resulting in host immune changes. For instance, functional reprogramming of macrophages during lipopolysaccharide tolerization was shown to be regulated by increased expression of miRNA-222, which targets BRG1 mRNA. BRG1 is the catalytic subunit of the chromatin remodeling SWI/SNF complex, and its decreased expression results in decreased transcription of the SWI/SNF-dependent late lipopolysaccharide-response genes with consequent macrophage tolerization [80]. Host microRNAs fine-tune macrophage activation by post-transcriptionally regulating signaling and metabolic genes involved in inflammation and resolution. Emerging evidence indicates that bacterial non-coding RNAs can enter host cells or alter host miRNA networks, highlighting cross-kingdom RNA-mediated regulation of macrophage gene expression and immune tone. miRNAs are small non-coding RNA molecules that regulate gene expression through mRNA degradation or translational repression. The interaction between the microbiota and host miRNA is bidirectional. The microbiota was shown to modulate the expression of the host miRNA, where the anerobic oral commensal *Fusobacterium nucleatum* activated Toll-like receptor 4 (TLR4) signaling with consequent activation of NF−κB and increased expression of miR2116. On the other hand, the host miRNA influenced the composition and activity of the microbiota. Several miRNAs were found to be differentially expressed in tissues from colorectal tumors and normal tissue, including the known oncogenic miRNAs miR-182 and miR-503 and the mir-17~92 cluster. These miRNAs were correlated with the relative abundances of several bacterial taxa, including Proteobacteria, Firmicutes and Bacteroidetes [81].

### 5.6. Epigenetic Control of Macrophage Polarization by Microbiota

The plasticity of macrophage polarization allows them to perform different roles in response to their environment. The gut microbiota metabolites could affect macrophage polarization, where the microbial fermentation product butyrate promoted M2 polarization and contributed to intestinal barrier integrity and anti-inflammatory effects. The mechanism of M2 polarization involved both HDAC inhibition and STAT6 activation, which are essential for the IL-4-mediated signaling response in M2 macrophage polarization [82]. On the contrary, lipopolysaccharides produced by Gram-negative bacteria were shown to be a potent activator of M1 polarization through activation of TLR4 signaling and NF-κB transcription with consequent transcriptional activation of pro-inflammatory genes like TNF-α and IL-6. Since macrophages play a crucial role in the pathogenesis of inflammatory bowel disease (IBD), the gut microbiota’s influence on macrophage polarization could control the progression of IBD, and the administration of probiotics could also induce a similar effect.

Nitrosylation can also have a multifactorial, context-dependent influence in macrophage polarization, serving as both an effector and a regulator. In M1 macrophages, inducible nitric oxide synthase (iNOS) is a major source of nitric oxide (NO), which functions as a reactive nitrogen species with inherent antitumorigenic and cytotoxic effects [11,13,14]. Additionally, it may function as a signaler, activating phagocytic processes, CCR5, and pro-inflammatory IL-12 [11,13,14]. Certain cancer types, such as prostate cancer, have been shown to downregulate NO through iNOS uncoupling, which leads to elevated oxidative stress states. The reintroduction of NO into these cell lines and subsequent S-nitrosylation of cysteine residues on the CSF1R oncogene resulted in decreased tumor burden, with increased M1 macrophages and decreased M2 macrophages [11,13,14]. However, other work suggests a role for the suppression of excessive M1 activation by nitration of tyrosine residues in IRF5, which blocks a classic M1 activation pathway [11,13,14]. These findings suggest that the complex role of nitrogen species in the TME is time- and context-dependent and can lead to both pro- and antitumorigenic effects within the framework of M1 and M2 polarization.

Glycosylation represents another layer of post-transcriptional modification that can influence macrophage polarization within the TME. One prominent example is hypersialylation, characterized by the addition of sialic acid-containing glycans to immune cells, which is associated with poor outcomes in human cancers [11,13,14]. Activation of Siglec receptors by hypersialylated ligands induces immunosuppressive signaling across multiple immune cell types, including the promotion of M2-like macrophage polarization and enhanced immune tolerance in murine models, whereas enzymatic removal of these ligands can restore pro-inflammatory M1 phenotypes [11,13,14].

N-glycosylation, or the attachment of a sugar molecule to the nitrogen of an asparagine residue, can also modulate macrophage expression. Players in the gut microbiota, such as Bacteroides spp., express carbohydrate-active enzymes (CAZymes) to degrade complex host N-glycans from sources like IgA [57,58,62,63]. Previous work suggests that inhibition of N-glycans on macrophages has been linked to increased phagocytosis and posits that N-glycosylation may skew towards a more protumorigenic, tolerant M2-like state [57,58,62,63]. Together, these data suggest that distinct glycosylation methods regulate macrophage polarization through divergent mechanisms, with significant implications for tumor immunity and microbiota–host interactions, warranting further investigation.

## 6. Therapeutic Implications

### 6.1. Therapeutic Implications of Microbiota in Associated Cancers

The recognition of the gut microbiota as a key determinant in cancer pathogenesis has catalyzed an emergent paradigm in oncologic research over the last decade. Gut microbiota composition, metabolites, and function have all been investigated for their diagnostic [33], prognostic [83,84], and prophylactic [85] relevance to therapeutic models. While the influence of the microbiota on tumor progression is well established, many therapeutic investigations have focused on microbiota-mediated modulation of the adaptive immune system, such as enhanced T cell activation and checkpoint inhibitor efficacy [86,87,88].

Studies continue to highlight the role of tumor-activated macrophages in the pathogenesis of various cancers [31]. As a dominant myeloid cell population within the TME with the capacity to either promote or suppress tumor progression depending on their polarization state [19], TAMs have emerged as a promising therapeutic target. Multiple experimental strategies—including TLR agonists, CD40 agonists, and PI3Kγ inhibitors—have demonstrated efficacy in reprogramming TAMs or limiting their tumor-supportive functions in preclinical and early-phase clinical trials [89].

Building on the growing recognition of TAMs as central regulators of the tumor microenvironment, recent clinical studies have established clear associations between TAM polarization states and patient outcomes. Specifically, an increased presence of M2-like, immunosuppressive TAMs correlates with greater tumor aggressiveness, angiogenesis, and resistance to therapy across multiple malignancies—including breast, lung, and colorectal cancers. Conversely, enrichment of M1-like, pro-inflammatory macrophages is linked to improved immune infiltration and favorable prognoses [48]. Abundant TAMs in patients with advanced-stage cancer had previously been associated with unfavorable clinical outcomes [90]; however, these findings have redirected interest from identifying strategies for depleting TAM populations to therapies that alter TAM phenotypes with therapeutic effect.

### 6.2. Gut Microbiota–TAM Axis

Recent findings have elucidated a mechanistic axis between the gut microbiota and TAMs, implicating this crosstalk as a key determinant of cancer outcomes. This gut microbiota–TAM axis is increasingly recognized for its ability to modulate TMEs through immunometabolic and signaling networks. Studies show that specific microbial-derived metabolites—such as SCFAs, secondary bile acids, and tryptophan catabolites—can influence TAM polarization and function via epigenetic reprogramming and engagement of pattern recognition receptors and nuclear receptors like TLRs, NOD-like receptors, and FXR/PXR pathways [91]. Harnessing this microbiota-driven mechanism of TAM polarization toward antitumor phenotypes presents an alternative and complementary strategy for altering the composition of the TME. Below, we examine the emerging strategies that leverage gut microbiota—via prebiotic, probiotic, and postbiotic interventions—to reprogram TAMs and enhance synergy with immunotherapies and epigenetic therapies.

### 6.3. Potential Risks of Butyrate and Postbiotic Use in Cancer Therapy

While butyrate is widely recognized for its anti-inflammatory and antitumorigenic properties [92], emerging evidence suggests that its use as a postbiotic therapy may pose risks in certain contexts. Notably, in prostate cancer models, elevated butyrate levels induced by fecal microbiota transplantation (FMT) or dietary intervention have been associated with increased tumor invasiveness and M2 macrophage recruitment [93]. These findings raise concerns about the long-term use of butyrate or SCFA-based therapies, especially outside the gastrointestinal tract. The pro- or antitumor effects of SCFAs appear to depend on dose, delivery method, tumor location, and local immune context [94]. A deeper understanding of the variables governing SCFA responses is essential to prevent unintended consequences such as immune suppression or enhanced metastasis in select tumor types.

Given the dual potential of microbiota-derived metabolites to either suppress [95] or support tumor growth [94,96], safety must be a key consideration in developing probiotic, prebiotic, and postbiotic therapies [97]. Personalized approaches—tailoring microbial strain selection, metabolite targeting, and delivery methods to tumor type and location—are essential. For example, therapies enhancing *A. muciniphila*-mediated STING activation may benefit immune-excluded tumors, while SCFA supplementation might be most effective in colorectal cancer [98]. Risks include unintended immune suppression [99], metabolic toxicity [100], and tumor-promoting epigenetic changes in off-target tissues [101]. Future research should aim to identify robust biomarkers for patient stratification and therapy monitoring to maximize efficacy while minimizing adverse outcomes [102,103].

### 6.4. Therapeutic Implications of Microbiotal Metabolites on Tumor-Associated Macrophages

Prebiotics are fermentable, non-digestible substrates that confer health benefits on the host by modulating the composition and function of commensal microorganisms [104]. Prebiotics are characterized by their ability to enrich specific microbiota (probiotics) whose metabolic outputs (postbiotics) have been observed to influence immune regulation, epithelial barrier integrity, and inflammatory tone [105]. In the context of cancer, these linked effectors have gained considerable attention for their capacity to shape the TME. Through downstream synthesis of microbial-derived metabolites such as short-chain fatty acids and cyclic dinucleotides, prebiotics and probiotics can cause effects on TAM polarization and function. Concurrently, they exert indirect effects by enhancing dendritic cell (DC) maturation, NK cell activation, and CD8^+^ T cell recruitment [105]—effectively reshaping the broader immune landscape in which TAMs operate.

This prebiotic–probiotic–postbiotic axis represents a network of interdependent pathways that converge on immune modulation [106], each level offering overlapping yet distinct opportunities for therapeutic intervention. Whether through altering microbial composition, enhancing functional metabolite production [107,108], or delivering purified microbial products, each strategy has demonstrated potential to influence macrophage phenotype and function within the TME.

The M2 polarization state of tumor-associated macrophages (TAMs) is not fixed and can be modulated under certain conditions [109,110]. Reprogramming strategies using HDAC inhibitors [111], STING agonists [112], or metabolic modulators (e.g., PPAR-γ inhibitors) [113] have shown promise in shifting macrophage phenotypes toward an M1-like, tumoricidal state. However, there are numerous factors that may limit the ability of these macrophage reprogramming approaches to shift TAMs to antitumor M1 macrophages. The extent and outcome of reprogramming may vary depending on tumor location, TAM plasticity, and the chronicity of exposure to immunosuppressive signals. Furthermore, therapies targeting M2 metabolism, such as inhibitors of fatty acid oxidation, must be carefully evaluated for systemic toxicity and off-target effects. Differences in macrophage behavior between tissues [114] highlight the need for precision approaches that consider the immunometabolic context of each tumor.

#### 6.4.1. Microbial Cyclic Dinucleotides and STING-Mediated TAM Reprogramming

A novel route has been identified through which prebiotic fiber augments host antitumor immunity via bacterial production of cyclic dinucleotides (CDNs) that engage the stimulator of interferon genes (STING) pathway in intratumoral myeloid cells. The STING pathway, defined by its ability to detect cytosolic DNA, initiates the transcription of pro-inflammatory cytokines in response to the presence of DNA within the cytoplasm [26603901]. CDNs are the naturally occurring agonists of STING and can originate from either microbial or host sources. Common bacterially synthesized CDNs include cyclic-di-AMP (cdAMP) and cyclic-di-GMP (cdGMP), while the CDN cyclic GMP-AMP (cGAMP) is produced by mammalian cells and associated with autoimmune disease [26603901]. Because clinically relevant CDNs are unique to either microbes or mammalian sources, their serum and fecal concentrations can be tracked and associated with microbiota-driven therapeutic outcomes. As a therapeutic device, STING activation has been well documented in reshaping the tumor microenvironment by inducing IFN-I responses, DC maturation, and priming of CD8^+^ T cells [115]. However, Lam et al. demonstrated that this pathway could be activated in mouse models with a pectin-rich diet, specifically identifying increased populations of *Akkermansia muciniphila* in the production of STING agonists that reprogrammed Ly6C^+^ monocytes toward immunostimulatory DC and NK cell-promoting phenotypes [116]. In this model, intratumoral IFN-I signaling driven by a microbial CDN, cdAMP, suppressed TAM-mediated immune tolerance by reprogramming TAMs to their M1-like counterparts. This effect was reproducible in germ-free mice following fecal microbiota transplantation (FMT) from high-fiber donors, underscoring the integral role of the bacteria *A. municiphila* in this prebiotic–probiotic–postbiotic axis.

Clinically speaking, the findings of Lam et al. have important implications for TAM-related cancer therapeutics. First, they establish a causal link and TAM-centered mechanism between prebiotics and myeloid cell fate in the TME. This diet-induced immunologic remodeling underscores the feasibility of using nutritional interventions to precondition the tumor microenvironment for improved therapeutic responsiveness. The *A. municiphila*-mediated reprogramming of monocytes was shown to significantly enhance tumor mass reduction of anti-PD-L1 therapy, suggesting a synergistic link between STING-dependent IFN-I activation and checkpoint blockade [116]. This, coupled with the transmissibility of these effects via FMT, suggest that some of the therapeutic benefits observed from FMT in immune checkpoint inhibitor (ICI) patients [117] may be TAM-mediated. Furthermore, Lam et al. found that systemic administration of the isolated postbiotic metabolite cdAMP elicited specific antitumor myeloid programming within the TME, suggesting that systemic administration of STING-agonist metabolites could be a more direct therapeutic avenue than prebiotic supplementation.

However, this approach comes with its own limitations, as studies have associated systemic administration of STING agonists with induction of severe dose-dependent cytokine-mediated adverse effects [118]. The short half-life and minimal membrane permeability of CDN STING agonists [37001564] necessitate high-dose administration to achieve effective antitumor concentrations—increasing the risk and lowering the effectiveness of systemic CDN–metabolite therapy. In order to safely utilize the immunomodulatory effects of STING activation in cancer therapy, further investigations must elucidate the role of STING in inflammation and autoimmunity. Studies have implicated chronic STING activation in the pathophysiology of autoimmune disorders, particularly those involving inflammatory responses to self-DNA (e.g., SLE, dermatomyopathies) [40552157]. Adding to the complexity, some research shows that STING deficiency alleviates symptoms in lupus-prone mice, while other studies suggest that STING activity may play a protective role by constraining TLR-mediated autoimmune responses [40552157]. A more recent study by Shibahara et al. found that elevations in dysbiosis-generated CDNs were correlated with increased IFN-I activity and anti-dsDNA antibodies in some, but not all, SLE patients [40334619]. While these studies reflect the poorly understood interplay of multifactorial autoimmune diseases, they also reinforce that STING modulation via the prebiotic–probiotic–postbiotic axis risks unintended cytotoxic or otherwise harmful effects outside of the tumor microenvironment target. To circumvent these off-target effects, novel therapeutic techniques—including antibody–drug conjugates of STING agonists and EGFR—are being investigated to increase tumor selectivity [118,119]. While this particular strategy does not allow for the direct use of microbial metabolites, it provides an example for how elucidating these effector molecules is being used to develop novel therapeutics.

#### 6.4.2. Tumor-Specific SCFA-Mediated Regulation of Myeloid Cells

Microbial-derived metabolites influence macrophage polarization through diverse and context-dependent pathways (Table 2). These bacterial taxa ferment complex carbohydrates that bypass digestion in the upper gastrointestinal tract, leading to increased colonic concentrations of SCFAs such as butyrate, acetate, and propionate [34]. The effects of SCFAs, particularly butyrate and propionate, upon TAM polarization are highly studied among the microbial metabolites, and recent findings underscore the tissue-specific effects of probiotics and postbiotics on TAM phenotypes and therapeutic outcomes.

In colorectal cancer models, butyrate supports M1-like, pro-inflammatory polarization by enhancing glycolysis and increasing reactive oxygen species, contributing to antitumor immunity [39]. This effect is mediated through histone acetylation (e.g., H3K9, H3K27), which facilitates chromatin accessibility and the transcription of antitumor effector genes [105]. Similarly, sodium butyrate has been shown to inhibit M2-like macrophages and reduce their immunosuppressive cytokines in murine melanoma models [39]. These antitumor effects demonstrate the capacity of butyrate to remodel the TME into a more immunostimulatory state.

However, there are limitations to the application of butyrate in the broad treatment of cancer, as the impact of butyrate is not universally beneficial. In prostate cancer models, fecal microbiota transplantation (FMT) from patients elevated luminal butyrate and acetate, which accelerated tumor progression [39]. Mechanistically, butyrate promoted autophagy and upregulation of the chemokine CCL20 via TLR3 signaling, fostering M2 macrophage recruitment and an immunosuppressive microenvironment [39].

Further complicating the picture, Duan et al. found that butyrate enhances oxidative phosphorylation and mitochondrial metabolism in macrophages, suppressing glycolysis by downregulating HIF-1α and enzymes such as HK2 and LDHA. This metabolic shift was associated with increased expression of M2 markers like *Arg1* and *IL-10* and reduced secretion of TNF-α and IL-6, indicating a more anti-inflammatory phenotype [39]. Specifically, butyrate acts as both an energy substrate and a signaling molecule, increasing mitochondrial respiration and ATP production while simultaneously inhibiting glycolysis through downregulation of HIF-1α and glycolytic enzymes such as HK2 and LDHA. This shift toward mitochondrial metabolism is associated with anti-inflammatory outcomes, including reduced secretion of TNF-α and IL-6, and increased expression of M2 markers such as Arg1 and IL-10 [39]. These findings challenge earlier assumptions that butyrate primarily supports M1 polarization via glycolytic activation and once again highlight the need to consider context and temporal dynamics when evaluating SCFA-induced metabolic phenotypes in TAMs.

Beyond SCFAs, other microbial metabolites contribute to macrophage functional polarization. Tryptophan metabolites, for example, signal through the aryl hydrocarbon receptor (AhR), promoting IL-10 production and M2-like differentiation. Secondary bile acids modulate macrophage activity through nuclear receptors including FXR and TGR5. Importantly, the immunologic effects of these metabolites are highly strain-dependent. *Faecalibacterium prausnitzii* and *Clostridium* spp. may promote M1 polarization in specific cancer settings [34], while *Akkermansia muciniphila* produces cyclic-di-AMP, which activates the STING pathway and enhances type I interferon (IFN-I) production, supporting an immunostimulatory macrophage phenotype.

Recent work by Nakkarach et al. further underscores the strain–tumor context relationship. Metabolites from certain *E. coli* strains suppressed tumor growth in lung and colorectal cancer models, but had no effect in breast or melanoma models [120]. This suggests that the therapeutic benefit of probiotics and postbiotics hinges on precise alignment between microbial strain, metabolite profile, and tumor type.

Together, these insights highlight the immense complexity and therapeutic potential of microbiota-based interventions in cancer. A comprehensive understanding of strain–metabolite–polarization relationships is essential for developing targeted strategies that modulate TAMs to improve patient outcomes. The broader microbiota–metabolite–immunity axis represents a promising, low-cost, and non-invasive adjunct to current oncologic therapies aimed at remodeling the tumor immune microenvironment. However, these findings also highlight the need for precision in the application of probiotics and postbiotics, where matching microbial strain, metabolite, and tumor context may be critical to realizing therapeutic benefit through TAM reprogramming.

## 7. Summary

Evaluation of solid tumors has shown that tumor-associated macrophages play a critical role in the tumor microenvironment and progression of malignancy. As an initial antitumor response, TAMs polarize to an M1 phenotype, exhibiting tumoricidal activity through the release of pro-inflammatory cytokines and reactive species to combat cancer cells. However, as tumors progress, a shift occurs towards an M2 phenotype, which fosters tumor growth and malignancy by supporting a protumor environment. M2-polarized TAMs contribute to tumor progression through various mechanisms, including promoting angiogenesis, secreting matrix-degrading enzymes, and engaging in feedback loops that enhance tumor invasion and metastasis. Additionally, M2 TAMs facilitate immune evasion by recruiting immunosuppressive cells and secreting inhibitory cytokines that dampen the activity of cytotoxic T cells and natural killer cells, allowing tumors to escape immune surveillance. For these reasons, an understanding of the signals that promote M2 polarization is useful.

The gut microbiota significantly influences macrophage polarization in the tumor microenvironment through the production of various metabolites, which modulate immune cell functions and metabolic pathways. Short-chain fatty acids, tryptophan metabolites, and bile acids play key roles in this interaction. SCFAs can promote pro-inflammatory macrophage polarization (M1) that supports antitumor immunity, while in other contexts, they may lead to anti-inflammatory (M2) macrophage polarization, showcasing their context-dependent effects. Tryptophan metabolites activate the aryl hydrocarbon receptor, enhancing immunosuppressive macrophage functions, while bile acids modulate inflammation and macrophage behavior through nuclear receptor activation.

Macrophage polarization is also tied to their metabolic state, with M1 macrophages favoring aerobic glycolysis and M2 macrophages relying on oxidative phosphorylation and fatty acid oxidation. These metabolic pathways are influenced by microbiota-derived metabolites, which in turn affect macrophage function, cytokine profiles, angiogenesis, and responses to therapies. Furthermore, the gut microbiota can impact tumor metabolism, and its dysregulation can lead to impaired immune responses.

Overall, microbiota-derived metabolites play a central role in regulating macrophage polarization and functioning in the TME, directly affecting tumor progression and therapeutic responses (Figure 2). Targeting the gut microbiota through dietary interventions, probiotics, or fecal microbiota transplantation represents a promising therapeutic strategy to enhance anti-cancer immunity and optimize cancer treatments. Additionally, profiling of the microbiome such as in the gastrointestinal tract may serve as a biomarker to stratify patients for optimal treatments, especially when considering response to immunotherapy [103].

## 8. Conclusions

The dynamic interplay between the gut microbiota and tumor-associated macrophages (TAMs) represents a critical axis in shaping the tumor microenvironment (TME) and influencing cancer progression. Through an array of microbial-derived metabolites, such as short-chain fatty acids, tryptophan catabolites, bile acids, and cyclic dinucleotides, the microbiota exerts both immunostimulatory and immunosuppressive effects on TAM polarization. These effects are highly context-dependent, varying by tumor type, metabolic state, cytokine milieu, and microbial strain.

Macrophage plasticity enables a reversible transition between pro-inflammatory (M1) and anti-inflammatory (M2) states, with profound implications for tumor immunity, angiogenesis, metastasis, and response to therapy. As such, the gut microbiota not only modulates immune surveillance and tumor tolerance but also emerges as a viable therapeutic target. Epigenetic modifications, PRR activation, and metabolic reprogramming are key mechanisms by which microbial signals rewire macrophage behavior.

Therapeutic strategies leveraging the microbiota—through prebiotics, probiotics, postbiotics, or targeted delivery of microbial metabolites—offer promising avenues to reprogram TAMs and synergize with existing immunotherapies. However, caution is warranted due to the potential for context-specific adverse effects, particularly in non-gastrointestinal tumors. Precision approaches that align microbial interventions with tumor characteristics, immune context, and metabolic landscape will be essential.

Altogether, this review highlights the pivotal role of the microbiota–macrophage axis in tumor biology and underscores the potential of microbiota-informed strategies to enhance cancer immunotherapy, reduce treatment resistance, and improve patient outcomes.

## Figures and Tables

**Table 1:** Distinct macrophage phenotypes and functions in the TME.

Phenotype	Inducing Signals	Key Markers/Effectors	Functions in TME	Transcriptional Regulators	Clinical Relevance
M1 (Classically Activated)	IFN-γ, TLR ligands (e.g., LPS)	IL-1β, IL-6, IL-12, IL-23, TNF-α, iNOS (NOS2), ROS, MHC-II, CD80/CD86	Tumoricidal, pro-inflammatory, enhances T-cell activation, promotes immune surveillance	STAT1, NF-κB	Associated with better prognosis; restrains early tumor growth
M2 (Alternatively Activated)	IL-4, IL-10, IL-13, TGF-β, CSF-1	IL-10, TGF-β, Arg-1, CD206, CD163, low IL-12	Immunosuppressive, promotes tissue remodeling, tumor growth, angiogenesis, and metastasis	STAT6, IRF4, PPAR-γ, STAT3	Correlated with poor prognosis; supports tumor progression and immune evasion
TAM (Tumor-Associated Macrophage)	TME-derived cytokines, hypoxia, adenosine, lactate, immune cell crosstalk	Mixed M1/M2 profile but mostly M2-like; PD-L1, CCL2, CCL22, MMPs	Early tumor: antitumor (M1-like); advanced tumor: protumoral, promotes invasion, metastasis, immune evasion	HIF-1α, HIF-2α, STAT3, PPAR-γ	High TAM infiltration (M2-like) = advanced stage, poor outcomes; potential therapeutic target
Plastic/Intermediate States	Dynamic environmental cues	Co-expression of M1 and M2 markers depending on local context	Functional adaptability; shifts between pro- and antitumor roles	Context-specific signaling integration	Reflects complexity of macrophages’ roles in vivo; target for macrophage reprogramming

**Table 2:** Microbial metabolites influencing macrophage polarization.

Metabolite	Microbial Source	Receptor(s) and Signaling Consequences	Mechanism	Macrophage Effect	Strain Examples
Butyrate	*Clostridium*, *Faecalibacterium*	GPR41 (FFAR3), GPR43 (FFAR2) → ↓ NF-κB, ↑ IL-10; SLC5A8 uptake → HDAC inhibition → chromatin relaxation	HDAC inhibition, TCA cycle shift	M1 or M2 (context-specific)	*Faecalibacterium prausnitzii*
Propionate	*Bacteroides*, *Akkermansia*	GPR41/43 → cAMP modulation; SLC5A8 → HDAC inhibition; promotes IL-10 and anti-inflammatory transcriptional programs	GPCR signaling, DNA methylation	M2-like	*Bacteroides thetaiotaomicron*
Tryptophan metabolites	*Lactobacillus*, *Bifidobacterium*	AhR → transcriptional suppression of NF-κB; ↑ IL-10; ↑ PD-L1; promotion of immunoregulatory TAM phenotypes	AhR activation	M2	*Lactobacillus reuteri*
Secondary Bile Acids	Gut microbiota conversion	FXR → ↓ NLRP3 inflammasome, ↓ IL-1β; TGR5 → ↑ cAMP–PKA → CREB activation → M2 polarization and metabolic shift toward OXPHOS	FXR, TGR5 signaling	M2	*Eubacterium*, *Clostridium* species
Cyclic di-AMP	*Akkermansia muciniphila*	STING → TBK1–IRF3 activation → IFN-I production → TAM reprogramming toward M1; ↑ DC/NK recruitment	STING pathway, IFN-I production	M1	*A. muciniphila*

**Table 3:** Epigenetic mechanisms modulated by microbiota in macrophages.

Epigenetic Mechanism	Microbial Influence	Effect on Macrophages/Host	Key Molecules/Pathways
DNA Methylation	Microbiota-derived B vitamins (e.g., folate, B12) and metabolites influence methyl donor availability (e.g., SAM)	Alters expression of protooncogenes; affects immune tolerance and cancer risk	SAM, c-fos, c-myc, TGF-β, Stat3
Histone Methylation	L-lysine stimulates SGOC metabolism, increasing SAM and histone methylation (e.g., H3K79me2)	Promotes immune tolerance via altered gene promoter methylation	H3K79me2, TGF-β, Stat3
Histone Acetylation	SCFAs like butyrate and propionate inhibit HDACs	Increases anti-inflammatory cytokines and antimicrobial peptides; promotes M2 polarization	HDACs, IL-10, antimicrobial peptides, STAT6
DNA Methylation (via SCFAs)	Propionate induces DNA methylation of DAB1 gene	Linked to metabolic outcomes such as vitamin D deficiency and diabetes risk	DAB1 promoter methylation
Chromatin Remodeling	Altered by microbial signals via miRNAs and ATP-dependent complexes	Affects accessibility of transcription factors; contributes to macrophage tolerization	BRG1, SWI/SNF complex, miR-222
miRNA Modulation	Microbiota regulates and is regulated by host miRNAs (e.g., miR-2116, miR-182)	Influences immune signaling and microbial composition; linked to tumorigenesis	TLR4, NF-κB, miR-2116, miR-182, miR-503, miR-17~92
Macrophage Polarization (M1/M2)	Butyrate promotes M2 polarization (anti-inflammatory); LPS promotes M1 polarization (pro-inflammatory)	Shapes immune responses in gut, affects IBD pathogenesis	HDAC inhibition, STAT6 activation, TLR4, NF-κB, TNF-α, IL-6
Nitrosylation	Gut microbiota bacterial nitrate reduction	Polarization to M1; Polarization to M2	CCR5 CSF1R/IRF5
Hypersialylation	Butyrate and propionate	Polarization to M2	Hexosamine biosynthetic pathway, Siglecs
N-glycosylation	Carbohydrate-active enzymes (CAZymes)	Decreased phagocytosis; Polarization to M2	CD32a, LAIR-1

## Data Availability

No new data were created or analyzed in this study.

## Abbreviations

A2A	Adenosine A2A Receptor
APC	Antigen-Presenting Cell
ATP	Adenosine Triphosphate
AhR	Aryl Hydrocarbon Receptor
B12	Cobalamin (Vitamin B12)
BRG1	Brahma-Related Gene 1 (catalytic subunit of SWI/SNF complex)
CCL	C-C Motif Chemokine Ligand
CD163	Cluster of Differentiation 163 (Scavenger Receptor)
CD206	Cluster of Differentiation 206 (Mannose Receptor)
CD80	Cluster of Differentiation 80
CD86	Cluster of Differentiation 86
CD8	Cluster of Differentiation 8 Positive T Cells
CREB	Cyclic AMP Response Element-Binding protein
CSF-1	Colony Stimulating Factor-1
CTLs	Cytotoxic T Lymphocytes
CVD	Cardiovascular Disease
DAMPs	Danger-Associated Molecular Patterns
EGF	Endothelial Growth Factor
EMT	Epithelial–Mesenchymal Transition
FFAR3	Free fatty acid receptor 3
FGF	Fibroblast Growth Factor
FMT	Fecal Microbiota Transplantation
FPR	Formyl Peptide Receptor
FXR	Farnesoid X Receptor
GM-CSF	Granulocyte-Macrophage Colony-Stimulating Factor
GPR	G-Protein-Coupled Receptor
H3K27ac	Acetylation of Histone H3 at Lysine 27
H3K79me2	Dimethylation of histone H3 at lysine 79
H3K9ac	Acetylation of Histone H3 at Lysine 9
HAT	Histone Acetyltransferase
HDAC	Histone Deacetylase
HIF	Hypoxia-Inducible Factor
IBS	Irritable Bowel Syndrome
ICIs	Immune Checkpoint Inhibitors
IRF4	Interferon Regulatory Factor 4
JAK-STAT6	Janus Kinase-Signal Transducer and Activator of Transcription 6 Pathway
LDHA	Lactate Dehydrogenase A
M0	Undifferentiated Macrophage State
M1	Classically Activated Macrophage
M2	Alternatively Activated Macrophage
MAMPs	Microbial-Associated Molecular Patterns
MAPK	Mitogen-Activated Protein Kinase
MDSCs	Myeloid-Derived Suppressor Cells
MHC-II	Major Histocompatibility Complex Class II
MMPs	Matrix Metalloproteinases
MTOR	Mechanistic Target of Rapamycin
NAD+	Nicotinamide Adenine Dinucleotide (oxidized form)
NF-kB	Nuclear Factor kappa-light-chain-enhancer of activated B cells
NLRP3	Nucleotide-Binding Oligomerization Domain-Like Receptor Protein 3
OXPHOS	Oxidative Phosphorylation
PD-L1	Programmed Death-Ligand 1
PDAC	Pancreatic Ductal Adenocarcinoma
PDGF	Platelet-Derived Growth Factor
PKA	Protein Kinase A
PPAR-gamma	Peroxisome Proliferator-Activated Receptor Gamma
PRRs	Pattern Recognition Receptors
PXR	Pregnane X Receptor
ROS	Reactive Oxygen Species
SAM	S-adenosyl-L-methionine
SAMe	S-Adenosyl Methionine
SCFA/SCFAs	Short Chain Fatty Acid(s)
SGOC	Serine, Glycine, One-Carbon metabolism
STAT1	Signal Transducer and Activator of Transcription 1
STING	STimulation of INterferon Genes
SWI/SNF	SWItch/Sucrose Non-Fermentable chromatin remodeling complex
Stat3	Signal Transducer and Activator of Transcription 3 gene
TAMs	Tumor-Associated Macrophages
TGF-beta	Transforming Growth Factor Beta
TGR5	G Protein-Coupled Bile Acid Receptor
TLR/TLR4	Toll-Like Receptor (4)
TLR2/TLR9	Toll-Like Receptor 2/9
TME	Tumor Microenvironment
TNF-alpha	Tumor Necrosis Factor Alpha
VEGF	Vascular Endothelial Growth Factor
XCI	X Chromosome Inactivation
cGAS	Cyclic GMP-AMP Synthase
cdAMP	Cyclic-di-AMP (Cyclic Diadenylate Monophosphate)
iNOS/NOS2	Inducible Nitric Oxide Synthase
uPA	Urokinase Plasminogen Activator
